# Creating Predictive Weed Emergence Models Using Repeat Photography and Image Analysis

**DOI:** 10.3390/plants9050635

**Published:** 2020-05-15

**Authors:** Theresa Reinhardt Piskackova, Chris Reberg-Horton, Robert J Richardson, Robert Austin, Katie M Jennings, Ramon G Leon

**Affiliations:** 1 Department of Crop and Soil Science, North Carolina State University, Raleigh, NC 27695-7620, USA; tareinha@ncsu.edu (T.R.P.); screberg@ncsu.edu (C.R.-H.); rob_richardson@ncsu.edu (R.J.R.); reaustin@ncsu.edu (R.A.); 2 Department of Horticulture, North Carolina State University, Raleigh, NC 27695-7609, USA; kmjennin@ncsu.edu

**Keywords:** emergence models, sigmoidal models, RGB, maximum likelihood analysis, supervised classification

## Abstract

Weed emergence models have the potential to be important tools for automating weed control actions; however, producing the necessary data (e.g., seedling counts) is time consuming and tedious. If similar weed emergence models could be created by deriving emergence data from images rather than physical counts, the amount of generated data could be increased to create more robust models. In this research, repeat RGB images taken throughout the emergence period of *Raphanus raphanistrum* L. and *Senna obtusifolia* (L.) Irwin and Barneby underwent pixel-based spectral classification. Relative cumulative pixels generated by the weed of interest over time were used to model emergence patterns. The models that were derived from cumulative pixel data were validated with the relative emergence of true seedling counts. The cumulative pixel model for *R. raphanistrum* and *S. obtusifolia* accounted for 92% of the variation in relative emergence of true counts. The results demonstrate that a simple image analysis approach based on time-dependent changes in weed cover can be used to generate weed emergence predictive models equivalent to those produced based on seedling counts. This process will help researchers working on weed emergence models, providing a new low-cost and technologically simple tool for data collection.

## 1. Introduction

Identifying weed emergence patterns and germination requirements are important steps for understanding weed biology and for timely control [1,2]. Predicting weed emergence will facilitate more efficient weed management practices, such as improving the timing of weed scouting and implementation of control measures before weeds are too large and the risk of escapes increases [3].

Weed emergence studies are primarily conducted in areas with a dense known natural population or are artificially seeded with the weed of interest [1,3]. The emergence pattern over time is tracked by regularly counting all seedlings that have emerged and removing them so that they will not be counted again. Because of the time and specialization in weed identification needed to take these measurements, weed emergence data has been limited to small areas and few locations [4].

Recent improvements in remote sensing capabilities and digital image resolution have provided new research approaches in agricultural systems [5,6]. Remote sensing includes image collection and analysis from satellites, airplanes, and ground cameras. Interest in remote sensing and its utility for weed management has existed for some time [7,8], but recently the availability and affordability of unmanned aerial vehicles (UAVs) and improved computing power has renewed interest in image collection and processing [5].

If emergence information could be extracted from images and used to create models similar to those that are derived by physical counts, collecting large amounts of data from a wide geographical area using remote sensing to develop future models could be possible [9]. For example, time series of satellite imagery and repeat photography have been able to create community budburst and senescence models of large tracts of forest [10,11,12]. In those studies, the individual trees were not counted, but the overall change in signal from the vegetation was used to create the phenology models of the forest.

Especially at small stages, weed coverage may provide enough information to estimate emergence even if the image analysis does not identify distinct plants for counting. For instance, the MoDiCoVi algorithm, for distinguishing pixels attributed to monocots compared to dicots, used coverage ratios to determine fertilizer applications [13,14]. Weed coverage might be a useful indicator of weed population density magnitude if images are taken shortly after emergence. Weed emergence studies already need to be set up in areas with high-density populations of the weed of interest, and the weeds need to be removed each week. These two experimental components simplify the applicability of image collection for quantifying emergence because the signals from individual weed seedlings captured in the image will be mainly from the primary species in those areas.

Weed emergence models focus on the pattern of seedling emergence accumulation relative to the total emergence at the end of the season, even when seedling density varies [1,15]. Similarly, the change in weed coverage over time should provide the same pattern. We hypothesized that repeat images that track the accumulation of pixels associated with the signal generated by weed seedlings could be used to predict the same pattern of emergence as could be found in actual counts. Therefore, the objectives of this study were to (1) quantify emergence of weed species using images over time and (2) correlate those values to actual counts under field conditions.

## 2. Results

### 2.1. Comparison of Workflow

Three different image analysis workflows of increasing processing intensity (thresholding, supervised classification, and supervised classification with postclassification steps) were compared using a subset of the *R. raphanistrum* emergence data to find a suitable method for the larger dataset (Table 1). The relative cumulative pixels from each workflow of each plot over time were regressed with the relative cumulative emergence for those same plots. It was determined that the best method was supervised classification + postclassification steps because this approach minimized the number of false positives. Color thresholding severely overestimated weed seedling pixels throughout the season, especially on days when no weed seedlings were present, and this resulted in an underestimation of the emergence pattern. Comparatively, using supervised image classification + postclassification steps in ArcMap eliminated many false positives, but at high weed densities could result in false negatives. High numbers of false negatives overestimate the rate of emergence.

When the complete dataset for *R. raphanistrum* and *S. obtusifolia* emergence data was used, postclassification steps were often too rigorous, eliminating polygons that actually were weed seedlings and lowering the coefficient of determination (R^2^; Table 2) of the relationship between relative cumulative pixels and the corresponding relative cumulative emergence derived from true counts. Supervised classification alone, while still providing some false positives in the form of isolated pixels, provided a more consistent error that provided a better prediction of emergence of true counts (Table 2).

### 2.2. Using Images to Model R. raphanistrum Emergence

**Figure 1:**
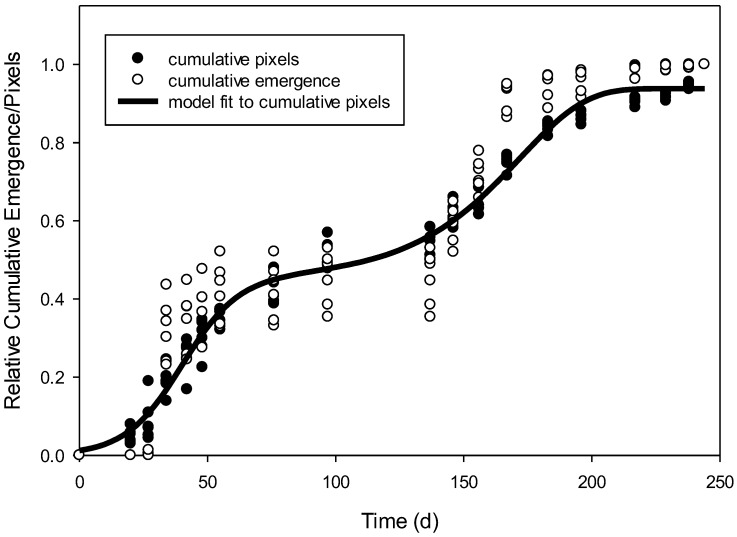
Relative cumulative pixels over time in days (dark circles). A biphasic equation was needed to fit the data as a predictive model for emergence of *Raphanus raphanistrum* (solid line). The relative emergence over time was based on true counts (white circles). None of the true count data were used to create the predictive model.

When the whole data set was used, relative cumulative pixels of *R. raphanistrum* using supervised classification provided a better prediction of relative cumulative emergence of true counts than supervised classification + postclassification (Table 2). Therefore, further mention of relative cumulative pixels refers only to pixels derived from the supervised classification method. The relative cumulative pixels of *R. raphanistrum* emergence followed a biphasic pattern, which required a two-phase model [16]. A sigmoidal + Weibull equation was fit to the relative cumulative pixels of *R. raphanistrum* over time in days (Figure 1). The predicted values of this model regressed with the observed cumulative pixels that were used to make the model resulted in R^2^ = 0.98 and a low root mean square error value (RMSE; Table 3). Additionally the Akaike’s information criterion (AIC), which is an index used to compare how well the model fit the data, is very negative. When this emergence model, which was generated only using the pixel information from images, was validated using the relative emergence of true counts, the model resulted in RMSE = 0.08 (Table 3). In other words, the RMSE indicated that the predictive model accounted for 92% of the variation in relative emergence derived from true counts.

### 2.3. Using Images to Model S. obtusifolia Emergence

**Figure 2:**
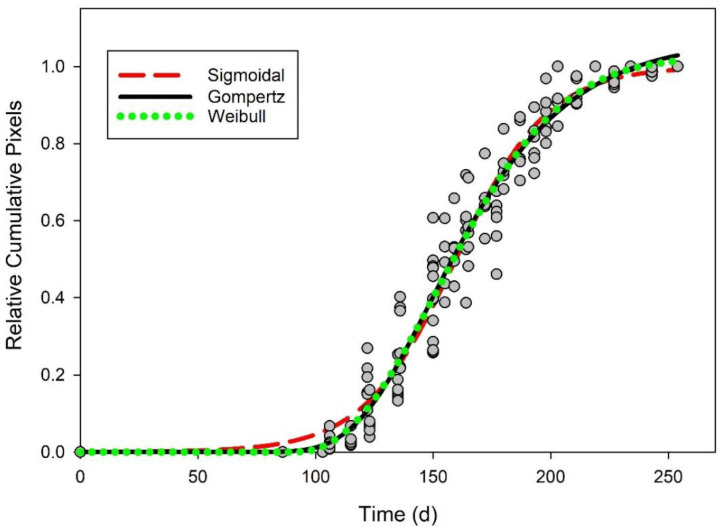
Relative cumulative pixels over time in days (gray circles). Three different equations were fit to the data as a predictive model for emergence of *Senna obtusifolia*: Sigmoidal (red dashed line), Gompertz (solid line), and Weibull (green dotted line).

**Figure 3:**
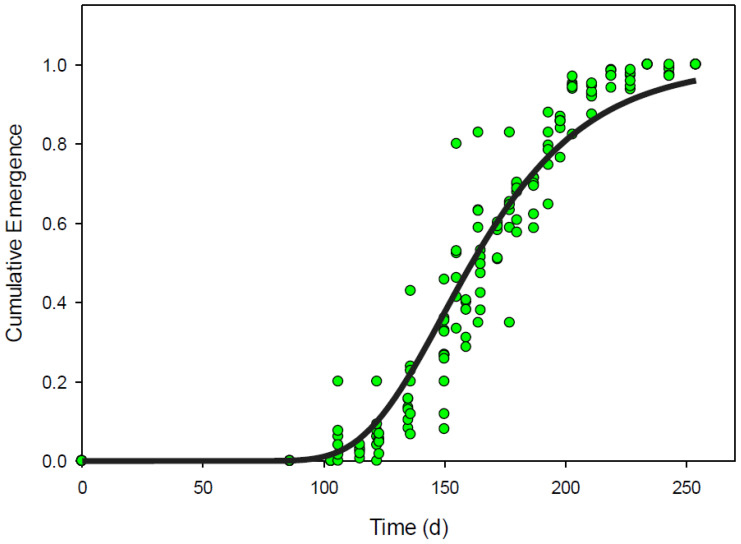
Expected *Senna obtusifolia* over time as predicted by the Gompertz model fit to relative cumulative pixels (solid line). Observed relative cumulative emergence from true counts of *Senna obtusifolia* over time (green circles).

Gompertz, simple sigmoidal, and Weibull equations could all be used to describe the pattern of emergence derived from images using relative cumulative pixels (Figure 2). Gompertz, sigmoidal, and Weibull equations all accounted for 93% of the variability (RMSE less than 0.07) in the cumulative pixel data and each had representative AIC values less than −430 (Table 4). Using the cumulative emergence from true counts for validation, these models accounted for more than 91% of the variation found in relative cumulative emergence compared to true counts (Table 4 and Figure 3).

## 3. Discussion

Using image analysis for seedling quantification is challenging because it is frequently assumed that image analysis cannot derive exact seedling numbers with accuracy [17,18]. The present study shows that even in the absence of seedling counts, seedling emergence patterns can be properly described by tracking changes in pixels associated with the weed of interest over time. Since *R. raphanistrum* seedlings have a somewhat uniform size, neighborhood analysis and polygon restrictions were expected to be needed to achieve this level of accuracy (Table 1); however, during times of high emergence and more leaf overlapping, the underestimation of weeds per image resulted in the overestimation of relative cumulative pixels at that date (Table 2). Over a longer period of time, the overestimation of relative cumulative pixels was more problematic than some underestimations of relative cumulative pixels using supervised classification. This is partly due to the conditions in which weed emergence data are collected. Primarily, site selection is based on the prevalent history for the weed of interest at high densities in the field. At lower weed densities, or with more nontarget weeds with distinctly different shape, the postclassification steps may improve the pixel enumeration overall; although, low density and mixed species sites would not be recommended for collecting data for weed emergence modeling regardless whether this is done with image analysis or seedling counts.

Recently, convolutional neural networks (CNNs, i.e., machine learning) were used to identify weed species based on RGB images [14,18,19]. Therefore, it is reasonable to expect that those systems might become valuable tools to quantify weed seedling emergence in the near future. However, those CNNs require thousands of images to train and test the system as well as high computing power to specifically process image information in a timely fashion [19,20]. Additionally, all of the work to this point was only done to identify plants larger than four true leaves [21,22]. The necessary high-density conditions and the frequency of weed quantification and removal for weed emergence modeling would require a new catalogue of weed seedling images to be developed for training based on cotyledon shape and size.

While we recognize that more advanced image analyses are being developed for weed identification, our approach provided an adequate description of seedling emergence patterns to achieve our modeling goals. Even so, there are many steps in our approach that could be automated for increased capacity. The steps of image processing in ArcMap can be programmed in Python and images processed in batches, which would eliminate the need to process the images individually. Another strategy to increase efficiency could be in setting up a stand with time lapse cameras paired with a timer to spray a broad-spectrum herbicide after the picture is taken. This would reduce the time for travel and data collection and the image processing since all images will have the exact same angle/area. The software could be adapted as well. Equivalent to ArcMap, Image J is an open source software that is accessible to all interested users.

## 4. Materials and Methods

### 4.1. Image Collection

Observation plots with 6 two-square meter quadrats were established in Clayton, NC, to monitor *Raphanus raphanistrum* L. emergence from September 2017 to May 2018 and in Kinston, NC, to evaluate *Senna obtusifolia* (L.) Irwin and Barneby emergence from April to August in 2018 and 2019. Field studies were set up in areas with a prevalent history of the weed of interest. No crop was planted in the study area for the duration of the experiment. Nontarget weeds, if any, were removed before images were taken. Quadrat-scale images were collected with a Cannon T5 from a height of 1.5 m with a resolution of 5184 × 3456 (18 MP) every two weeks at the beginning of the study and every three weeks when seedling emergence rate decreased. Photographs were taken in full sun or uniformly overcast days and within 2 hours of solar noon, minimizing the variability in color and shadows between images taken on the same day. After photographs were taken, weed seedlings were counted and removed (i.e., true counts). It must be emphasized that the aim of this work was not to provide evidence for the value of emergence modeling, but to test if simple image analysis procedures could be used to replace the manual labor of counts needed to create predictive emergence models.

### 4.2. Image Analysis

The primary goal of image processing was to code the image in a way that the number of pixels corresponded to the reflectance signal generated by the weed of interest. From there, the weed coverage could be determined for a given day based on pixels in the image, rather than the number of weeds themselves. The challenge was to find an appropriate method to achieve accurate classification of the weed pixels apart from other distracting soil features commonly found when the images are taken at high resolution close to the ground.

A preliminary study was conducted with the first 54 images of *R. raphanistrum* emergence (September to December) to find a suitable method for analyzing each image. The first attempt to elucidate weed emergence coverage from the images was done with an open source image editing and analysis package called GIMP (v.2.8.22; www.gimp.org 1997–2019). Because the quadrats were cleaned of nontarget weed species before photographing, it appeared that weeds were green tissue on bare soil background; however, at high ground resolution, other soil features can have shades of green that the human brain will ignore due to context [23]. Images were brought into the software, clipped to the borders of the quadrat, and then a binary image was created by a process called thresholding [24]. First, the contrast in the image was exaggerated (this contrasted green pixels from the nongreen) and then a spectral value was set, where pixels above were considered nonweed, and pixels below considered to be the weed. This thresholding procedure highlighted the weeds, but also included many nonweed pixels. In the preliminary subset of 54 images, regression of relative emergence based on seedling counts with relative cumulative pixels by the thresholding method resulted in R^2^ = 0.76 (Table 1).

Another software with more analytical functions, ArcMAP (v10.5.1, Environmental Systems Research Institute, Redlands, CA), was used to allow pixel-based spectral classification. Supervised classification using maximum likelihood analysis statistically clusters pixels of the image into user defined categories, also called labels. The labels were defined by highlighting sections (training samples) of one image that represented those labels; this was used as the reference file. Once the reference file was created from one image, it was used to classify other images that contained the same features. In other words, the software determined the likelihood of each pixel belonging to any of the defined labels based on the similarity to spectral values in the reference file. Henceforth, this process is referred to simply as “supervised classification”.

White panels are often used to calibrate colors before analyzing images in order to remove the variations in light quality between images [21]. Even when using white panels, the colors within an image need to be consistent for the calibration to be effective; this was accomplished by taking pictures during full sun or full cloud cover. Additionally, to reduce shadows on full sun days, the pictures were taken within 2 hours of solar noon. The white panels were insufficient in accounting for the differences in light quality, so it was necessary to use separate reference files for sunny days and overcast days. One reference file defining 5 labels with 20 training samples each was created to represent cloudy days and one reference file defining 6 labels with 20 training samples each was created to represent sunny days. On cloudy days, the distinct labels were “weed seedling”, “sand”, “soil”, “sticks”, and “rocks” and on sunny days, a class for “shadows” was added as a sixth label.

For each image, a supervised classification was run with one of the aforementioned reference files depending whether the image was from a day that was cloudy or sunny. The supervised classification procedure used training samples for several categories and returned the total number of pixels assigned to each label. The supervised classification with training samples of multiple labels reduced the misclassification of pixels, compared to the thresholding approach performed in GIMP. Within the preliminary subset, the regression of relative emergence with relative cumulative pixels using the supervised classification method resulted in R^2^ = 0.86 (Table 1).

Some postclassification steps were tried to account for size. Both *R. raphanistrum* and *S. obtusifolia* were relatively large seedlings, therefore, any isolated pixels labeled “weed” were frequently false positives. To correct for this, a moving window or neighborhood analysis was run with a majority filter. Using circle neighborhoods of 15-pixel radius eliminated isolated pixels that were not weeds and helped group neighborhood pixels. This classified raster was converted to polygons and then the areas of individual polygons could be used to eliminate shapes that were too large to be a weed seedling. The regression between relative emergence and the relative cumulative pixels using supervised classification with postclassification on the preliminary subset resulted in R^2^ = 0.99 (Table 1).

The results of this preliminary comparison of methods using a subset of the data led us to the conclusions that (1) thresholding was not adequate to derive the weed data from each image to create emergence models and (2) supervised classification and postclassification steps were necessary to obtain the most accurate information from each image for creating an emergence model. However, since supervised classification was a step in the process, we were able to quantify the model improvement added by the postclassification steps.

A total of 174 and 114 images were classified for *S. obtusifolia* and *R. raphanistrum*, respectively. Each processed image resulted in total pixels and pixels labeled “weed”. These were used to convert each image into a value that represented weed coverage in the picture: the proportion of pixels labeled as the weed of interest, based on the total pixels in the image for each evaluation. This proportion was converted back into a pixel number for a standard-size image of 12,500,000 pixels, because the classified area of pictures varied, but had an average of 12,846,459 pixels. The labeled pixels for each image of the same quadrat over time were summed for each day and then converted to relative cumulative pixel accumulation for that quadrat over the entire season.

Initially, the selection of methods (above) was decided by regressing the relative cumulative emergence (from true counts) with the relative cumulative pixels resulting from the series of image processing steps. The methods described were thresholding, supervised classification, and supervised classification with postclassification steps. Regression was done using PROC REG in SAS 9.4 (SAS Institute Cary, NC, USA). The R^2^ value demonstrating the strength of each relation is described in Table 1. In order to test if image processing would provide a model similar to true counts, further analysis was needed. Relative cumulative pixels over time were used to fit sigmoidal models using SigmaPlot 14.0 (Systat Software Inc. San Jose, CA, USA). Model parameters were tested using PROC NLMIXED in SAS and the Akaike information criterion (AIC) was used for determining how well the model fit the data. The expected values of the model were also regressed with the relative cumulative emergence from true counts to determine how well the emergence from true counts was predicted by the model (derived from pixel data). Low RMSE (approaching zero) and high R^2^ values (approaching one) indicate a strong prediction by the fitted model and describe the accuracy of using images to create predictive models compared to the actual counts.

## 5. Conclusions

Supervised classification of RGB images using a relative few training samples and images to generate signature files were enough to provide a classification of emerged seedlings; no hyperspectral or multispectral cameras were needed. Weed emergence data collection has been stymied by the limited time and funding available for doing this type of research. The time for collecting and processing these data could be reduced by using RGB images automatically analyzed by supervised classification and thus, increasing the number of researchers willing or able to participate in data collection. Weed emergence and even phenology modeling might be possible using technology that is available now, to model changes in vegetation over time. While machine learning technologies might be within sight, it will still take time to develop the necessary catalogues of weed seedling images, and once available, will not necessarily benefit all countries where weed predictive models could improve management timing. The approach used in the present research could provide an easy and affordable way to collect weed emergence data without the need for large image databases that include multiple species. This will allow more researchers to collaborate and develop weed emergence models over a wider geographical, technological, and budgetary range.

## Figures and Tables

**Table 1:** Comparison of relative cumulative emergence based on seedling counts and relative cumulative pixels using three different image analysis methods. Observations and images of *R. raphanistrum* emergence from September to December were used, totaling 54 comparisons.

Image Classification Method	RMSE	R^2^
Binary color thresholding	0.20	0.76
Supervised classification	0.15	0.86
Supervised classification + postclassification	0.04	0.99

**Table 2:** Comparison of the relationship between relative emergence from true counts and the relative cumulative pixels achieved by two methods (supervised classification alone and in combination with postclassification).

Species	Method	R^2^
*R. raphanistrum*	supervised classification	0.95
	supervised classification + postclassification	0.54
*S. obtusifolia*	supervised classification	0.92
	supervised classification + postclassification	0.84

**Table 3:** Predictive model of *R. raphanistrum* emergence fit to relative cumulative pixels over time. Predicted values were regressed with observed pixel values used to create the model (RMSE and R^2^) and for validation, with relative cumulative emergence of true counts (RMSE validation).

Model	Equation	AIC ^ab^	RMSE	R^2^	RSME Validation
Sigmoidal + Weibull	$y=\frac{0.4553}{1+e^{-\left(\frac{x-42}{13}\right)}}+\left(1-0.4831\right)\left(1-e^{-{\left|\frac{x-162.7+ln2^{\frac{1}{744991}}}{19000000}\right|}^{744991}}\right)$	−413	0.04	0.98	0.08

^a^ AIC is the Akaike’s information criterion used for comparing models. The more negative values are better fit. ^b^ AIC, RMSE, and R^2^ reflect the fit of the model with the relative cumulative pixels, used to create the model. ^c^ validation was done by comparing the predictive models, based on pixels, with corresponding relative emergence data based on weed seedling counts.

**Table 4:** Possible predictive models of *S. obtusifolia* emergence fit to relative cumulative pixels over time. Predicted values were regressed with observed pixel values used to create the model (RMSE and R^2^) and for validation, with relative cumulative emergence of true counts (RMSE validation).

Model	Equation	AIC ^ab^	RMSE	R^2^	RSME Validation
Gompertz	$y=1e^{\left(-e^{-\left(\frac{x-171}{30}\right)}\right)}$	−448	0.066	0.96	0.085
Sigmoidal	$y=\frac{1}{1+e^{-\left(\frac{x-787}{210}\right)}}$	−436	0.068	0.96	0.084
Weibull	$y=\left(1\right)\left(1-e^{-{\left|\frac{x-159+ln2^{\frac{1}{2.2}}}{80}\right|}^{2.2}}\right)$	−440	0.065	0.96	0.086

^a^ AIC is the Akaike’s information criterion used for comparing models. The more negative values are better fit. ^b^ AIC, RMSE, and R^2^ reflect the fit of each model with the relative cumulative pixels, used to create the model. ^c^ validation was done by comparing the predictive models based on pixels with corresponding relative emergence data based on weed seedling counts.
